# Characteristics of referred muscle pain to the head from active trigger points in women with myofascial temporomandibular pain and fibromyalgia syndrome

**DOI:** 10.1007/s10194-012-0477-y

**Published:** 2012-08-31

**Authors:** Cristina Alonso-Blanco, César Fernández-de-las-Peñas, Ana Isabel de-la-Llave-Rincón, Pedro Zarco-Moreno, Fernando Galán-del-Río, Peter Svensson

**Affiliations:** 1 Department of Nursing, Universidad Rey Juan Carlos, Alcorcón, Madrid Spain; 2 Department of Physical Therapy, Occupational Therapy, Rehabilitation and Physical Medicine, Universidad Rey Juan Carlos, Alcorcón, Madrid Spain; 3 Esthesiology Laboratory of Universidad Rey Juan Carlos, Alcorcón, Spain; 4 Department of Rheumatology, Fundación Hospital Alcorcón, Alcorcón, Spain; 5 Department of Clinical Oral Physiology, School of Dentistry, University of Aarhus, Aarhus, Denmark; 6 Department of Oral and Maxillofacial Surgery, Aarhus University Hospital, Aarhus, Denmark; 7 Orofacial Pain Laboratory, Center for Sensory-Motor Interaction, Aalborg University, Aalborg, Denmark; 8 Facultad de Ciencias de la Salud, Universidad Rey Juan Carlos, Avenida de Atenas s/n, 28922 Alcorcón, Madrid Spain

**Keywords:** Temporomandibular disorders, Fibromyalgia, Trigger points, Referred pain, Pain assessment

## Abstract

Our aim was to compare the differences in the prevalence and the anatomical localization of referred pain areas of active trigger points (TrPs) between women with myofascial temporomandibular disorder (TMD) or fibromyalgia (FMS). Twenty women (age 46 ± 8 years) with TMD and 20 (age 48 ± 6 years) with FMS were recruited from specialized clinic. Bilateral temporalis, masseter, sternocleidomastoid, upper trapezius, and suboccipital muscles were examined for TrPs. TrPs were identified by palpation and considered active when the pain reproduced familiar pain symptom experienced by the patient. The referred pain areas were drawn on anatomical maps, digitalized and also measured. A new analysis technique based on a center of gravity (COG) method was used to quantitative estimate of the localization of the TrP referred pain areas. Women with FMS exhibited larger areas of usual pain symptoms than women with myofascial TMD (*P* < 0.001). The COG coordinates of the usual pain on the frontal and posterior pain maps were located more superior in TMD than in FMS. The number of active TrPs was significantly higher in TMD (mean ± SD 6 ± 1) than in FMS (4 ± 1) (*P* = 0.002). Women with TMD exhibited more active TrPs in the temporalis and masseter muscles than FMS (*P* < 0.01). Women with FMS had larger referred pain areas than those with TMD for sternocleidomastoid and suboccipital muscles (*P* < 0.001). Significant differences within COG coordinates of TrP referred pain areas were found in TMD, the referred pain was more pronounced in the orofacial region, whereas the referred pain in FMS was more pronounced in the cervical spine. This study showed that the referred pain elicited from active TrPs shared similar patterns as usual pain symptoms in women with TMD or FMS, but that distinct differences in TrP prevalence and location of the referred pain areas could be observed. Differences in location of referred pain areas may help clinicians to determine the most relevant TrPs for each pain syndrome in spite of overlaps in pain areas.

## Introduction

Temporomandibular (TMD) pain is a musculoskeletal local pain condition with a prevalence rate between 3 and 15 %, an incidence rate between 2 and 4 % [1] and a women–men ratio 2:1 [2]. Similar, fibromyalgia (FMS) is a widespread diffuse musculoskeletal pain condition with a prevalence ranging from 0.5 % to 5.0 and a higher proportion of women [3, 4]. Although the etiology and pathology of TMD and FMS are under debate, there is evidence of facilitated nociceptive processes in both conditions. In fact, TMD pain has been frequently diagnosed on fibromyalgia subjects [5, 6] whereas the presence of widespread pain has been shown to be a significant risk factor for myofascial TMD [7]. A recent study has found an increased risk for the onset of clinically significant TMD pain when subjects also had FMS [8]. Therefore, it seems that TMD and FMS may share some patho-physiological mechanisms.

In the last years, there has been an increasing interest for the role of myofascial trigger points (TrPs) in both TMD [9] and FMS [10]. TrPs are defined as hypersensitive spots within a taut band of a skeletal muscle that are painful on stimulation and give rise to a referred distant pain [11]. Active TrPs are spots with local and referred pain that reproduce the clinical pain symptom experienced by the patient and associated with the usual pain reported by the patient. Latent TrPs have similar clinical findings as active TrPs, but, in contrast, they do not mimic the painful symptoms [11]. Clinical distinction between active and latent TrPs is substantiated by histo-chemical findings because higher levels of neuroactive mediators (i.e., bradykinin, substance P, or serotonin) have been found in active TrPs compared with latent TrPs and non-TrP [12, 13].

Two recent studies have demonstrated that referred pain from TrPs in the head and neck muscles reproduced symptoms in women with myofascial TMD [14] or FMS [15]. Nevertheless, these previous studies did not provide a quantitative estimation of the localization of referred pain areas. Two previous studies have used a new analysis technique based on a centre of gravity (COG) method to estimate the localization of the referred pain areas elicited by injection of hypertonic saline into head and neck muscles in healthy subjects [16, 17]. With this technique it would be possible to estimate the anatomical localization of the referred pain elicited by active TrPs in pain populations. Previous studies demonstrated that FMS is highly co-morbid with primary headaches such as tension-type and migraine headaches [18, 19] and TMD [5–7]. The identification and quantification of differences in TrP distribution and location of the referred pain between patients with TMD or FMS could be helpful for better clinical identification of pain patterns in these populations in spite of overlaps in symptomatology and clinical presentation of pain. In addition, identification of those muscles most affected by TrP in each group of patients could possibly also be helpful for better management of TrP in these populations. Application of the COG technique with quantification of overlapping pain patterns in the head could therefore be an important novel feature in a more standardized description of patients with TMD and FMS.

To the best of the authors’ knowledge, no previous study has investigated the differences in the prevalence of active muscle TrPs and the anatomical localization of the referred pain areas between patients with TMD and FMS. Therefore, the aims of the current study were to compare the differences in the prevalence and the anatomical localization of referred pain areas of active TrPs in head and neck–shoulder musculature between women with strict myofascial TMD and FMS.

## Methods

### Participants

In the current study, women with a main diagnosis of FMS or myofascial TMD were included. Participants were recruited among patients referred from a tertiary specialized clinic on chronic pain in Madrid, Spain. They were carefully screened and explored to further determine the inclusion and exclusion criteria by an experienced dentist (TMD group) or rheumatologist (FMS group). All patients had prior to the referral received treatment but without adequate pain relief, i.e., they represented, at least in part, a treatment resistant proportion of myofascial TMD and FMS patients.

To be included in the TMD group, women should fulfill a primary and exclusive diagnosis of myofascial TMD according to the Research Diagnostic Criteria for TMD (RDC/TMD) [20]. Pain location, range of jaw motion, temporomandibular joint (TMJ) pain, clicking sounds, crepitation, and pain upon palpation of muscles and TMJ were assessed with the use of the RDC/TMD criteria. Pain should be presented with duration of at least 3 months and an intensity of at least 3 on an 11-point numerical pain rating scale (NPRS; 0: no pain; 10: maximum pain). Subjects were excluded if they exhibited any of the following: 1, signs or symptoms according to categories II-III of the RDC-TMD criteria (disc displacement, arthralgia, osteoarthrosis or osteoarthritis); 2, TMJ surgery or steroid injections; 3, comorbid fibromyalgia; 4, diagnosis of any systemic disease (rheumatoid arthritis, systemic lupus erythematosis, psoriatic arthritis); 5, previous cervical or head trauma; 6, presence of a score >8 points in the BDI-II; 7, diagnosis of primary headache (tension-type headache or migraine); or, 8, cognitive impairment.

To be included within the FMS group, women should fulfil the criteria from the American College of Rheumatology (ACR) [21]. Tender points were tested by digital palpation at the nine paired sites according to the ACR by an experienced rheumatologist. The presence of widespread pain, fatigue and altered sleep was also recorded. In this group, the presence of TMD symptoms was not considered as exclusion criteria. Subjects were excluded if presented any of the following: 1, severe physical disability; 2, comorbid conditions (inflammatory disease, irritable bowel syndrome, interstitial cystitis); 3, uncontrolled endocrine disorders (hypo-thyroidism, diabetes); 4, diagnosis of primary headaches (tension-type headache or migraine); 5, malignancy; 6, psychiatric illnesses (schizophrenia or substance abuse); 7, medication usage other than as-needed analgesics (excluding long-term narcotics); 8, history of surgery; 9, previous history of whiplash injury; 10, presence of a score >8 points in the BDI-II; or, 11, cognitive impairment.

The study protocol was approved by local Ethics Committee (URJC 08-30) and conducted following the Helsinki Declaration. All participants signed informed consent prior to their inclusion.

### Self-reported measures

**Fig. 1:**
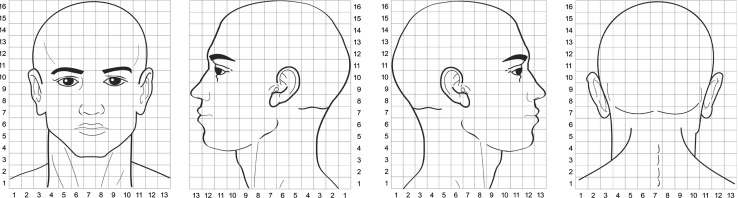
Schematic presentation of the center-of-gravity (COG) technique. The *X* and *Y* coordinates of the COG were calculated in a 13 × 16 grid system (see “Methods”)

A numerical pain rate scale (NPRS) was used to assess the usual level of pain, worst and lowest level of pain experienced in the preceding week at rest in the orofacial area [22]. In the present study, the usual level of pain was related to pain experienced normally by the patient, and not the pain level on the day of examination. Women with FMS were asked to focus the pain assessment on the orofacial region. They were also asked to draw the distribution of their usual pain pattern within an anatomical map including lateral, frontal, and occipital projections of the face (dimensions 65 × 80 mm, Fig. 1).

The Beck Depression Inventory (BDI-II) a 21-item self-report measure assessing affective, cognitive and somatic symptoms of depression, was used to exclude patients with depressive symptoms (BDI-II >8 points) [23]. The BDI-II has shown good internal consistency (α = 0.86) with higher scores indicating higher levels of depression [24, 25].

### Muscle trigger point (TrP) examination

Trigger points were bilaterally explored within the temporalis, masseter, upper trapezius, sternocleidomastoid and suboccipital muscles by an examiner with more than 10 years’ experience in muscle TrP examination. TrP diagnosis was done following the criteria as described by Simons et al. [11]: 1, presence of a palpable taut band in a skeletal muscle; 2, presence of a sensitive spot within the taut band; 3, local twitch response elicited by the snapping palpation of the taut band; and 4, presence of referred pain in response to TrP compression (approximately 20 N force for 5 s). Gerwin et al. [26] found that these criteria, when applied by an experienced examiner, have good inter-examiner reliability (kappa) ranging from 0.84 to 0.88.

Trigger point diagnosis within the suboccipital muscles was made when there was local tenderness in the suboccipital region, referred pain with maintained pressure for 10 s and increased referred pain with active extension of the upper cervical spine [27].

Trigger points were considered active when both the local and the referred pain evoked by the compression reproduced the usual pain symptoms, and the elicited pain was familiar for the participant [11]. For that purpose, after TrP assessment on each muscle, subjects were asked: “When I pressed this muscle, did you feel pain or discomfort locally, and in another area (referred pain). If yes, please tell me whether the pain that you felt in the other area reproduced the pain that you normally experience.” The order of muscle TrP evaluation was randomized between participants. As both groups have pain symptoms, TrP assessment was conducted in a blinded fashion in relation to specific diagnosis.

### Assessment of referred pain area and center of gravity (COG)

Local pain was defined as pain located around the compression site, and referred pain was defined as the pain located at least 1 cm outside the local pain area evoked by TrP palpation. Participants were asked to draw the distribution of the referred pain after palpation of each muscle TrP on the same anatomical map including lateral, frontal, and occipital projections of the face/neck (dimensions: 65 × 80 mm). Specific information on possible referral patterns was avoided in order not to induce bias [28]. The usual pain area and the TrPs referred pain areas were measured with a digitizer (ACECAD, model D9000 + digitizer, Taiwan) to calculate the area of perceived pain expressed in arbitrary units (au).

To obtain a quantitative estimate of the localization of the perceived pain areas, usual pain symptoms or referred pain areas elicited by active TrPs, the centre-of-gravity (COG) was calculated according to previous studies [16, 17]: a grid outline with 5-mm resolutions (i.e., a total of 13 × 16 = 208 grids) was superimposed on lateral, frontal and occipital pain maps (Fig. 1). Each grid in the coordinate system was assigned a value on a dichotomous basis (0: no pain, 1: pain). The COG coordinates (*X* anterior–posterior, *Y* inferior–superior) in arbitrary units (au) were calculated according to the following formula:

$$ \begin{gathered} x = \sum_{i = 0}^{13} \sum_{j = 0}^{16} \left( {x_{i} \times {\text{grid value}}_{i,j} } \right) / \sum_{i = 0}^{13} \sum_{j = 0}^{16} {\text{grid value}}_{i,j} \hfill \\ y = \sum_{i = 0}^{13} \sum_{j = 0}^{16} \left( {y_{i} \times {\text{grid value}}_{i,j} } \right) /  \sum_{i = 0}^{13} \sum_{j = 0}^{16} {\text{grid value}}_{i,j} \hfill \\ \end{gathered} $$

### Statistical analysis

Results are expressed as means, standard deviations (SD) and 95 % confidence intervals (95 % CI). The Kolmogorov–Smirnov test revealed a normal distribution of all quantitative variables (*P* > 0.05). Differences in pain parameters between groups were assessed with the unpaired Student’s *t* test. The Chi square (χ^2^) test was used to assess the differences in the distribution of TrPs for each muscle on either side within both groups. A two-way analysis of variance (ANOVA) with area (frontal, non-dominant dominant, posterior) as within-subjects variable and group (TMD, FMS) as the between-subjects variable was used to assess differences in usual symptomatic pain areas between groups. Differences in *X* and *Y* coordinates of usual pain areas between groups were assessed with the unpaired Student’s *t* test. Differences in the number of active TrPs between groups were assessed with the non-parametric Mann–Whitney *U* test. A three-way analysis of variance (ANOVA) was used to compare referred pain areas (au) between sides (dominant/non dominant) and muscles (i.e., temporalis, masseter, upper trapezius, and sternocleidomastoid) as within-subjects factors and group (TMD, FMS) as between-subjects factor. A similar 2-way ANOVA was used for the referred pain area from suboccipital muscles without side as factor. The Bonferroni test was used for post hoc analyses. Differences in *X* and *Y* coordinates of TrPs referred pain areas between groups were assessed with the unpaired Student’s *t* test. Finally, the Spearman’s rho (*r*_s_) test was used to analyze the association between the number of TrPs, the referred pain areas and pain parameters. A *P* value < 0.05 was considered statistically significant.

## Results

### Demographic and clinical data of the sample

Table 1 summarizes clinical and demographic data of both groups. Sixty-two (*n* = 62) consecutive patients with a diagnosis of TMD (*n* = 30) or FMS (*n* = 32) were screened for eligibility criteria. Ten (33 %) patients with TMD [migraine (*n* = 4), fibromyalgia syndrome (*n* = 4), previous whiplash (*n* = 2)] and 12 (37 %) with FMS [high levels of depression (*n* = 7), previous whiplash (*n* = 5)] were excluded. Finally, a total of 20 women with strict myofascial TMD (mean age 46 ± 8 years) and 20 women with FMS (age 48 ± 6 years) satisfied all the inclusion criteria and agreed to participate.. Women with FMS exhibited a longer duration of the pain condition (*P* < 0.001), and higher usual level of pain, worst and lowest level of pain experienced in the preceding week at rest in the orofacial area (*P* < 0.001).

**Table 1:** Clinical pain parameters between women with myofascial TMD (*n* = 20) or FMS (*n* = 20)

	Myofascial TMD	FMS	Significance
Pain history (years)	2.3 ± 1.3 (1.7–2.9)	6.7 ± 2.3 (5.6 –7.8)	*z* = −5.003; *P* < 0.001
Usual level of pain (0–10)	4.1 ± 1.3 (3.5–4.8)	6.0 ± 1.0 (5.5–6.4)	*z* = −3.812; *P* < 0.001
Worst level of pain (0–10)	6.5 ± 0.9 (6.0–6.9)	8.4 ± 0.8 (8.0–8.8)	*z* = −4.787; *P* < 0.001
Lowest levels of pain (0–10)	1.9 ± 0.8 (1.6–2.4)	3.8 ± 1.0 (3.3–4.3)	*z* = −4.538; *P* < 0.001

Data are expressed as mean ± SD (95 % confidence interval)

*TMD* temporomandibular disorder, *FMS* fibromyalgia syndrome, *NPRS* numerical pain rate scale

### Usual pain areas in the orofacial region

Table 2 shows the mean values of *X* and *Y* coordinates of the COG for women with myofascial TMD or FMS. The mean usual pain area in women with myofascial TMD was 12.5 au (95 % CI 8.1–17.0) in the frontal region (*n* = 17, 85 %), 43.7 au (95 % CI 28.4–59.0) in the occipital region and posterior part of the neck (*n* = 12, 60 %), 24.5 au (95 % CI 17.3–31.7) on the dominant side of the head (*n* = 14, 70 %), and 17.8 au (95 %CI 12.1–23.6) in the non-dominant side of the face (*n* = 20, 100 %). Women with FMS reported a usual pain area of 36.1 au (95 % CI 22.0–49.8) in the frontal area (*n* = 19, 95 %), 71.1 au (95 % CI 46.3–95.9) in the occipital region and posterior part of the neck (*n* = 20, 100 %), 34.3 au (95 % CI 25.1–43.6) on the dominant side of the head (*n* = 16, 80 %), and 27.2 au (95 % CI 18.5–36.4) on the non-dominant side of the face (*n* = 15, 75 %).

**Table 2:** Center-of-gravity (COG) measurements of usual pain drawing in the orofacial region in women with myofascial TMD or with FMS [mean ± SD (95 % CI)]

	Frontal projection		Posterior projection		Dominant (right) side		Non-dominant (left) side	
	*X*	Y*	*X*	Y*	*X*	*Y*	*X*	*Y*
TMD	7.4 ± 1.2 (6.7–7.2)	10.4 ± 2.3 (12.5–13.5)	6.9 ± 0.4 (6.8–7.0)	7.1 ± 2.3 (5.7–7.6)	7.3 ± 1.2 (7.4–8.9)	9.5 ± 2.0 (12.0–14.0)	7.3 ± 1.6 (7.5–8.2)	8.7 ± 1.8 (11.8–13.4)
FMS	6.8 ± 0.7 (6.4–7.0)	12.1 ± 2.9 (13.5–14.0)	7.1 ± 0.2 (7.0–7.1)	5.3 ± 1.2 (6.2–10.5)	7.4 ± 1.1 (6.0–8.0)	11.3 ± 3.4 (11.5–14.6)	7.4 ± 2.1 (6.9–8.5)	8.9 ± 4.4 (12.4–14.0)

*TMD* temporomandibular disorder, *FMS* fibromyalgia syndrome

* Significant differences between TMD and FMS (Student’s *t* test, *P* < 0.05)

The ANOVA indicated significant differences between groups (*F* = 15.207; *P* < 0.001) and regions (*F* = 12.005; *P* < 0.001) for usual pain symptoms. No significant group × region interaction was found (*F* = 1.005, *P* = 0.370). The post hoc analysis revealed that women with FMS exhibited larger pain areas of usual pain symptoms in the face than women with myofascial TMD (*P* < 0.01), and that the pain area within the posterior region of the head was significantly larger than the remaining pain areas (*P* < 0.001). No significant associations between the intensity or duration of pain and usual pain areas in either group were found (*P* > 0.145).

**Fig. 2:**
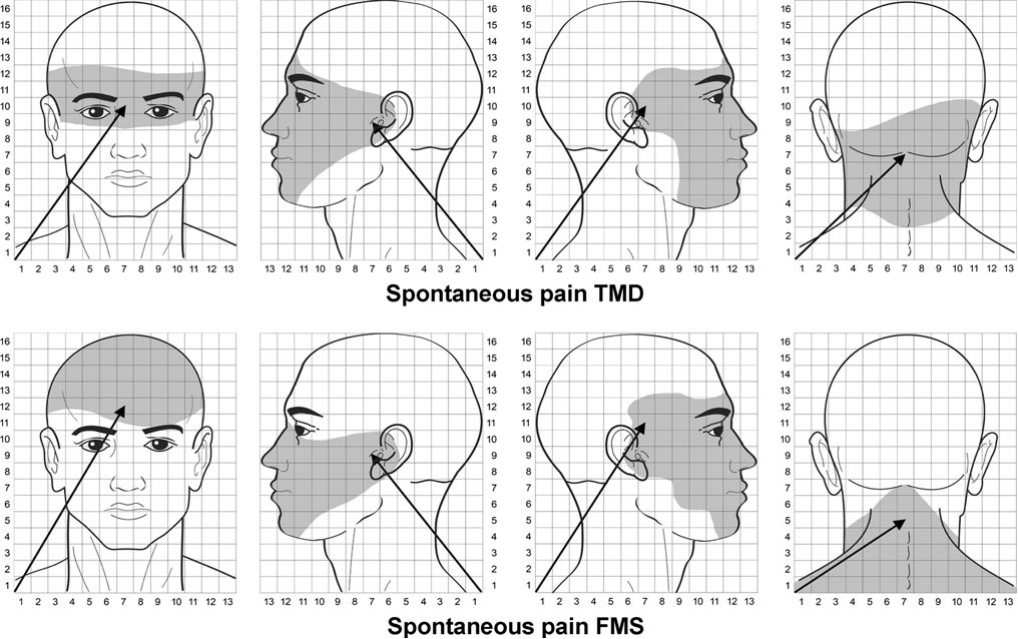
Center-of-gravity (COG) and areas of the usual pain symptoms in women with TMD (*top*) or with FMS (*bottom*). The length of the pain vector (*arrow*) is computed in arbitrary units

In addition, significant differences between the location of the *Y* coordinate on the frontal side (*t* = −2.190; *P* = 0.045) and posterior region (*t* = −2.981; *P* = 0.006): the pain areas in women with TMD were located more superior (higher *Y* value) than in women with FMS. No significant differences for the remaining coordinates of the COG were found between groups (*P* > 0.147, Fig. 2).

### Prevalence of Muscle TrPs

Table 3 details the distribution of TrPs in both women with myofascial TMD or FMS. The mean ± SD number of active TrPs in women with myofascial TMD was 6 ± 1 and 4 ± 1 in women with FMS (*z* = −3.105; *P* = 0.002). A significant association between the number of active TrPs and usual pain (*r*_s_ = 0.492; *P* = 0.027) was found in TMD, but not in FMS (*r*_s_ = 0.027; *P* = 0.909). No other association between the number of active TrPs and the other pain parameters was found.

**Table 3:** Number of women with myofascial temporomandibular disorder or with fibromyalgia with active muscle trigger points (TrPs) in head and neck–shoulder muscles

	Temporalis*		Masseter		Upper trapezius		Sternocleidomastoid*		Suboccipital
	Right	Left	Right	Left*	Right	Left*	Right	Left	Bilateral
Myofascial Temporomandibular disorders (*n* = 20)									
Active TrPs (*n*)	12	16	12	16	12	16	8	11	14
No TrPs (*n*)	8	4	8	4	8	4	12	9	6
Fibromyalgia syndrome (*n* = 20)									
Active TrPs (*n*)	5	1	14	10	18	11	3	5	13
No TrPs (*n*)	15	19	6	10	2	9	17	15	7

* Significant differences between TMD and FMS (Chi square, *P* < 0.05)

The distribution of active muscle TrPs between women with myofascial TMD or FMS was significantly different for both temporalis (right: χ^2^ = 18.649, *P* < 0.001; left: χ^2^ = 26.471, *P* < 0.001), left masseter (χ^2^ = 6.154, *P* = 0.046), left upper trapezius (χ^2^ = 10.326, *P* = 0.006), and both sternocleidomastoid (right: χ^2^ = 21.073, *P* < 0.001; left: χ^2^ = 12.939, *P* = 0.002) muscles: women with myofascial TMD exhibited a greater number of active TrPs in these muscles as compared to women with FMS.

No significant differences in the distribution of right masseter (χ^2^ = 4.732, *P* = 0.984), right upper trapezius (χ^2^ = 5.700, *P* = 0.058), and suboccipital (χ^2^ = 4.637, *P* = 0.098) muscles were found between groups.

### TrP referred pain areas

Table 4 summarizes size of the referred pain areas in all muscles in women with myofascial TMD or FMS. A three-way ANOVA revealed significant differences in referred pain areas between muscles (*F* = 24.002, *P* < 0.001), but not between groups (*F* = 3.023, *P* = 0.083) or sides (*F* = 0.084, *P* = 0.772). A significant interaction between group × muscle (*F* = 4.601, *P* = 0.004), but not for group × side (*F* = 0.009, *P* = 0.926), side × muscle (*F* = 0.030, *P* = 0.993) or group × muscle × side (*F* = 0.061, *P* = 0.980) was found.

**Table 4:** Referred pain areas (au) of active trigger points (TrPs) in head and neck–shoulder muscles in women with myofascial temporomandibular disorder or with fibromyalgia

Myofascial temporomandibular disorder		Fibromyalgia syndrome	
Temporalis			
Right side (*n* = 12)	20.8 ± 10.8 (14.6–27.2)	Right side (*n* = 5)	19.1 ± 8.5 (12.4–25.7)
Left side (*n* = 16)	21.2 ± 11.2 (14.5–27.9)	Left side (*n* = 1)	19.3 ± 9.9 (12.8–25.8)
Masseter			
Right side (*n* = 12)	14.5 ± 6.3 (8.1–21.3)	Right side (*n* = 14)	16.5 ± 11.3 (9.8–23.1)
Left side (*n* = 16)	15.4 ± 5.4 (9.0–22.3)	Left side (*n* = 10)	14.6 ± 6.4 (8.1–21.0)
Upper trapezius			
Right side (*n* = 12)	36.1 ± 18.2 (29.4–42.7)	Right side (*n* = 18)	31.5 ± 19.0 (25.1–38.0)
Left side (*n* = 16)	35.3 ± 16.1 (28.8–41.7)	Left side (*n* = 11)	30.8 ± 21.6 (24.3–37.2)
Sternocleidomastoid*			
Right side (*n* = 8)	7.1 ± 3.8 (5.9–13.3)	Right side (*n* = 3)	24.8 ± 13.9 (18.1–31.4)
Left side (*n* = 11)	4.3 ± 3.2 (2.8–9.9)	Left side (*n* = 5)	25.7 ± 14.6 (18.9–32.5)
Suboccipital*			
Bilateral (*n* = 14)	7.6 ± 3.4 (5.8–11.1)	Bilateral (*n* = 13)	49.6 ± 29.1 (38.6–60.6)

* Significant interaction between group × muscle (3-way ANOVA test, *P* < 0.01)

The two-way ANOVA for the suboccipital muscles revealed significant differences between group (*F* = 30.646, *P* < 0.001) and muscle (*F* = 17.391, *P* < 0.001) and an interaction between group × muscle (*F* = 15.438, *P* < 0.001). The Bonferroni analyses showed that women with FMS exhibited larger referred pain areas than those with myofascial TMD for sternocleidomastoid and suboccipital muscles (*P* < 0.001), but not for the temporalis, masseter or upper trapezius (*P* > 0.763). The referred pain area elicited by suboccipital TrPs was significantly larger than the referred pain elicited from the remaining muscles (*P* < 0.001) within the FMS, but smaller within the TMD (*P* < 0.01). Further, the referred pain area from upper trapezius TrPs was significantly larger than the referred pain areas elicited by temporalis, masseter and sternocleidomastoid muscle TrPs in both groups (*P* < 0.001).

**Fig. 3:**
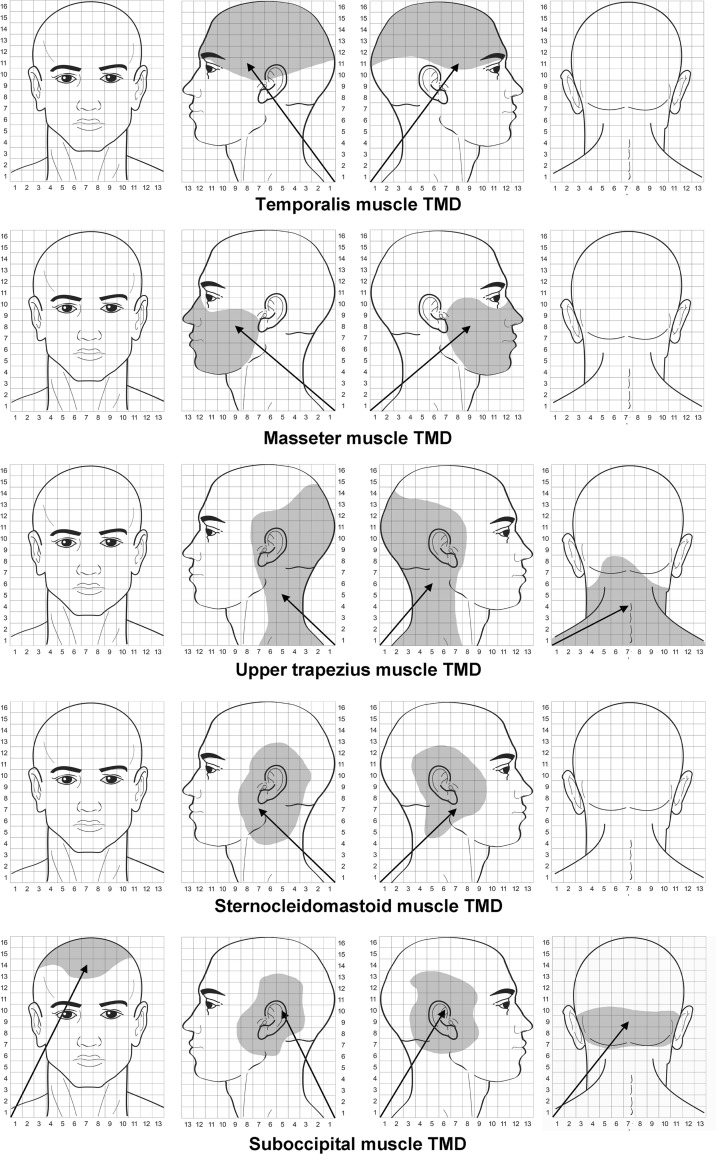
Center-of-gravity (COG) and areas of the referred pain elicited by active TrPs in women with myofascial TMD. The length of the pain vector (*arrow*) is computed in arbitrary units

**Fig. 4:**
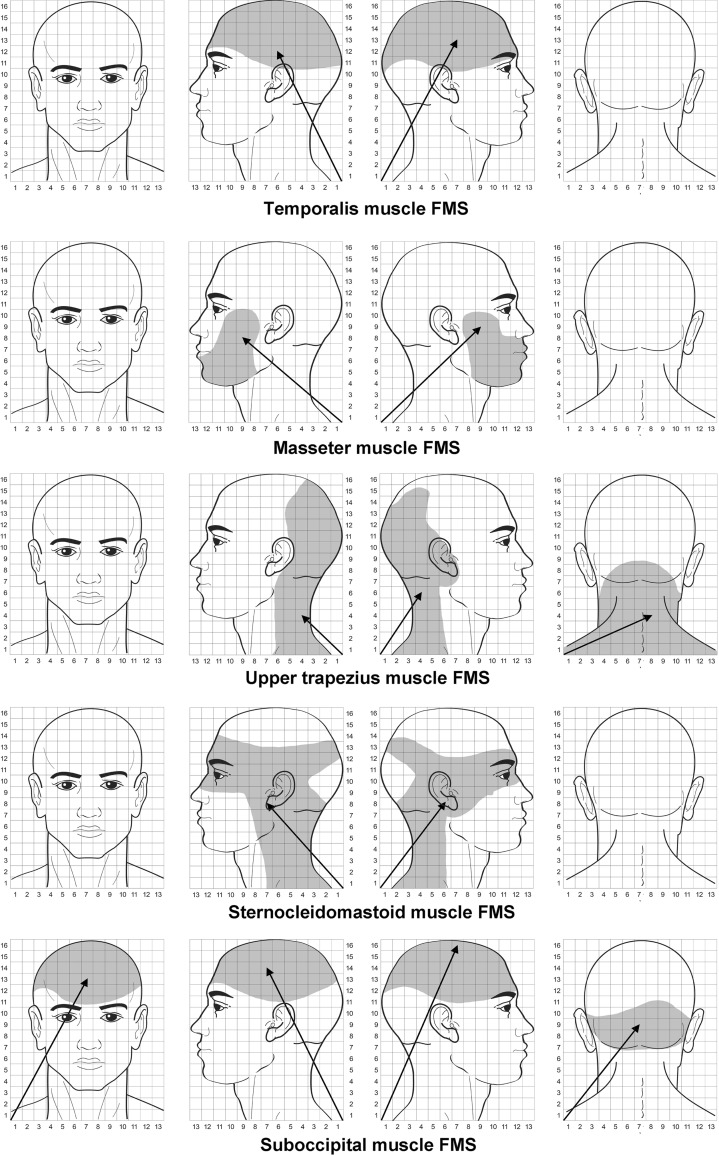
Center-of-gravity (COG) and areas of the referred pain elicited by active TrPs in women with FMS. The length of the pain vector (*arrow*) is computed in arbitrary units

Table 5 summarizes mean values of *X* and *Y* coordinates of the COG of referred pain areas from TrPs for women with TMD or TMD. Significant differences between *Y* coordinates within the temporalis (*t* = −2.929, *P* = 0.008) and masseter (*t* = −2.921, *P* = 0.007) muscles of the right (dominant) side were found: the TrP referred pain area in women with TMD (Fig. 3) was located more inferior (lower *Y* values) than in women with FMS (Fig. 4). Significant differences were also found within the upper trapezius muscle for *X* coordinates of the posterior area (*t* = −2.320, *P* = 0.027) and *X* (*t* = −2.015, *P* = 0.048) and Y (*t* = −2.243, *P* = 0.035) coordinates on the left (non-dominant) side: TrP referred pain area in the posterior part of the head was located more medially (greater *X* value) in women with FMS (Fig. 4) than in women with TMD (Fig. 3), whereas the referred pain area of the left upper trapezius was located more medially (greater *X* value) and superior (greater *Y* value) in women with TMD. Finally, referred pain areas from suboccipital muscle TrPs were more superior (greater *Y* value) bilaterally (*t* = −2.407, *P* = 0.037) in women with TMD (Fig. 3) than in those with FMS (Fig. 4).

**Table 5:** Center-of-gravity (COG) measurements of referred pain drawing in women with myofascial temporomandibular disorder or with fibromyalgia

	Frontal projection		Occipital projection		Dominant (right) side		Non-dominant (left) side	
	*X*	*Y*	*X*	*Y*	*X*	*Y*	*X*	*Y*
Temporalis muscle								
TMD	NA	NA	NA	NA	7.7 ± 1.1 (7.1–8.3)	11.5 ± 1.4* (10.8–12.3)	7.6 ± 0.8 (7.2–8.0)	11.0 ± 1.4 (10.3–11.7)
FMS	NA	NA	NA	NA	7.2 ± 1.4 (5.8–8.6)	13.3 ± 0.5 (12.8–13.9)	6.1 ± 1.0 (5.7–9.9)	12.0 ± 1.4 (8.6–14.7)
Masseter muscle								
TMD	NA	NA	NA	NA	8.9 ± 1.0 (8.4–9.4)	7.9 ± 1.1* (7.4–8.5)	9.0 ± 0.8 (8.6–9.4)	7.6 ± 1.2 (7.0–8.2)
FMS	NA	NA	NA	NA	9.1 ± 0.8 (8.6–9.5)	9.4 ± 1.6 (8.5–10.3)	9.3 ± 0.8 (8.6–9.9)	8.3 ± 1.3 (7.3–9.4)
Upper trapezius muscle								
TMD	NA	NA	6.6 ± 1.9* (5.6–7.7)	4.1 ± 1.5 (3.3–4.9)	4.6 ± 1.1 (4.0–5.3)	5.6 ± 1.5 (4.8–6.4)	4.5 ± 1.1* (3.8–5.2)	5.2 ± 1.7* (4.3–6.2)
FMS	NA	NA	8.1 ± 1.6 (7.2–8.9)	4.4 ± 0.9 (3.8–4.9)	4.1 ± 0.4 (4.0–4.3)	5.8 ± 0.9 (5.4–6.3)	3.8 ± 0.4 (3.5–4.1)	3.9 ± 0.9 (3.3–4.6)
Sternocleidomastoid muscle								
TMD	NA	NA	NA	NA	7.0 ± 0.8 (6.6–7.5)	6.8 ± 1.9 (5.8–7.8)	7.2 ± 0.7 (6.8–7.5)	6.6 ± 2.0 (5.7–7.6)
FMS	NA	NA	NA	NA	5.5 ± 1.6 (3.5–7.4)	8.6 ± 3.3 (6.3–12.9)	6.7 ± 1.3 (5.1–8.3)	8.3 ± 1.9 (5.9–10.8)
Suboccipital muscles								
TMD	7.1 ± 0.4 (6.5–7.7)	13.7 ± 1.9 (10.7–16.7)	7.0 ± 0.5 (6.7–7.3)	8.9 ± 1.2 (8.3–9.6)	5.5 ± 2.5 (3.8–7.1)	10.4 ± 2.2* (8.9–11.9)	5.3 ± 2.0 (4.0–5.7)	10.4 ± 2.2* (9.1–11.8)
FMS	6.9 ± 0.1 (6.8–7.3)	13.3 ± 1.2 (12.1–14.6)	7.1 ± 0.3 (6.9–7.3)	9.7 ± 2.8 (7.6–11.8)	7.0 ± 0.3 (6.8–7.2)	16.1 ± 0.5 (16.0–16.5)	7.0 ± 0.7 (5.6–12.3)	14.5 ± 0.7 (8.1–18.8)

*NA* not applicable (there was no referred pain to this area of the head)

* Significant differences between women with myofascial TMD and FMS (Student’s *t* test, *P* < 0.05)

## Discussion

This study revealed that the referred pain elicited from active TrPs in head and neck–shoulder musculature reproduced the pain pattern in the orofacial region in women with myofascial TMD or FMS. In addition, differences in TrP prevalence and spreading of the referred pain areas were observed as women with myofascial TMD exhibited more active TrPs than women with FMS, whereas women with FMS exhibited larger referred pain areas for sternocleidomastoid and suboccipital muscles. Furthermore, the referred pain areas of temporalis and masseter muscle TrPs were located more inferior (within the orofacial area) in women with TMD, whereas upper trapezius TrP referred pain was located more inferior (located in the neck area) in women with FMS.

The relationship between orofacial pain and FMS has been previously reported as it is commonly seen that women with FMS also exhibit symptoms in this area [5, 6]. In the current study, we found that women with FMS exhibited a longer duration of the painful condition and higher levels of pain in the orofacial area than those with myofascial TMD, which is to be expected as FMS is a chronic condition with longer period before the diagnosis. Another difference was that usual pain within the frontal and occipital areas was more superior (higher *Y* value) in women with TMD than in women with FMS. Further, women with FMS exhibited larger areas of usual pain than women with TMD, which is expected as FMS is characterized by a greater impairment of nociceptive pathways [8]. The present findings reveal that the location of usual pain symptoms in the orofacial region is similar between women with pure myofascial TMD and FMS, but the pain is more widespread in FMS.

The presence of active TrP referred pain in myofascial TMD or FMS corroborates previous studies, which have demonstrated the relevance of active muscle TrPs in women with myofascial TMD [14] or FMS [15, 29] as compared to healthy controls. In the current study, TrPs in the masticatory muscles, temporalis and masseter, were more prevalent than TrPs in neck muscles, sternocleidomastoid and suboccipital, in the TMD group whereas the opposite was found in women with FMS. Our findings are in contrast with data from tension-type headache where TrPs in the cervical muscles were the most prevalent [30–32]. It is expected that masticatory muscle TrPs play a more relevant role in orofacial pain, whereas neck and shoulder TrPs could play a greater role in headaches. In fact, TrPs in the neck muscles were more prevalent than in the masticatory muscles in women with FMS, possibly reflecting clinical differences in muscle pain generators between women with TMD or FMS. This distinction has potential important clinical implications as it has been suggested that regional pain in FMS, in this case orofacial pain, can be caused by local active TrPs and/or referred from remote active TrPs. A previous study showed that the overall usual FMS pain is not simply diffuse body pain but is located to certain body areas [33] supporting this hypothesis. Therefore, the current study supports the hypothesis that pain generators in different muscles can be involved in orofacial pain (TMD), neck pain associated to FMS and tension-type headache. Nevertheless, clinicians should be aware that treatment of TMD and FMS may vary but could in some patients include TrP therapy. In fact, recent studies have supported the clinical relevance of TrPs in both TMD [14] and FMS [15] related pain. It is possible that a proper therapeutic approach targeted to specific TrPs in the muscles identified by the described COG technique in the current study may improve clinical outcomes in these populations. Further studies are, however, needed to establish the efficacy of TrP therapy in TMD and FMS.

In addition, clinical differences between TMD and FMS were also present in the location of referred pain areas as temporalis and masseter TrPs referred pain to the head (more superiorly) in FMS but to the face (more inferiorly) in TMD. In addition, suboccipital TrPs referred pain was also more superiorly located in the head in TMD than in FMS (neck). Therefore, it seems that TrP referred pain in FMs is mostly located in the neck region, whereas TrP referred pain in TMD is mostly located in the face area. One possible patho-physiological mechanism explaining differences in the location of the TrP referred pain can be related to a different clinical manifestation or degrees of trigeminal brain stem sensory nuclear complex sensitization relative to cervical/spinal sensitization. It is plausible that women with TMD would exhibit a greater sensitization of the trigeminal neurons than women with FMS which would explain the TrP referred pain location in the face rather than in the neck for TrPs in the masticatory muscles.

The presence of active TrPs indicates sensitization of muscle nociceptors in both TMD and FMS since high levels of neuroactive mediators [12, 13] and lower pressure pain thresholds [34–37] have been found in active TrPs. In fact, Hong [38–40] suggested the concept of multiple sensitized nociceptors in the TrP region as the sensory units responsible for the local twitch response when the TrP is mechanically stimulated. Further, there is evidence demonstrating that active TrPs may serve as potent peripheral noxious input sensitizing central nervous system in FMS and TMD. This hypothesis is supported by human experimental studies showing that a single intramuscular anesthetic injection into the midpoint of the upper trapezius muscle, a typical site of active TrPs in FMS patients, significantly increased pain thresholds and decreases secondary heat hyperalgesia in FMS [41]. Therefore, studies investigating the clinical relevance of TrPs inactivation in the course of pain in the orofacial area in women with myofascial TMD or FMS are clearly needed.

Finally, we should recognize some strengths and limitations of the study. First, we included patients recruited from tertiary clinics which may not represent the general population. It is possible that patients seeking treatment in specialized clinics exhibit different features and characteristics than those recruited from a general population and it is a common clinical observation that these patients have inadequate pain relief from a variety of therapeutic approaches (e.g., oral appliances, medication, etc.) [42]; so direct extrapolations of the current results to the general population should be avoided at this stage. In addition, our sample size was relatively small. Therefore, future studies with greater sample sizes and including patients recruited from the general population are now needed to further confirm the current results. Furthermore, FMS diagnosis is a challenge for clinicians and researchers and new diagnostic criteria have been proposed [43]. Women with FMS included in our study were diagnosed according to first diagnostic criteria [21]. We do not know if the same results would be found in women with FMS presenting the new proposed criteria, but we speculate that there is a significant concordance between the two sets of criteria. Second, the TrP examination was conducted by a blinded examiner ruling of the chance of bias. Nevertheless, it is possible that a potential bias of patients’ recognition of the referred pain may be present in TrP examination although this seems unlikely. In addition, reproducibility of TrP diagnosis has been questioned in recent reviews since studies of high quality are needed [44, 45]. Factors that may have contributed to the varying reliability of the results from previous studies are lack of identification of taut bands, inexperience of the examiners in assessing TrPs or incorrect palpation techniques. Nevertheless, Gerwin et al. [26] reported that TrP diagnosis has good inter-examiner reliability when applied by an experienced examiner, as this was conducted in this study. It should also be acknowledged that the understanding of TrP remains incomplete and further studies will be needed to clarify underlying mechanisms as well as clinical manifestations. Third, we included a specific group of women with strictly myofascial TMD or with FMS. We excluded patients with other concomitant RCD/TMD diagnosis and other co-morbid conditions, i.e., primary headaches, with may be also related to the presence of TrPs. Therefore, our results should be considered with caution when extrapolate to different groups of patient with FMS or TMD. Fourth, it is suggested that psychological factors, e.g., anxiety and depression, may enhance central nervous system responses [46]. In the current study, we excluded a state of depression in our patients with TMD or FMS (>8 points BDI-II). Therefore, data are representative for women with myofascial TMD pain or FMS without depression. Nevertheless, we do not know if anxiety, sleep disturbances, pain catastrophizing etc. could also influence our results. Finally, since we only included women, future studies should investigate gender differences and determine if similar results are present in men with TMD or FMS.

## Conclusion

This study showed that the referred pain elicited from active TrPs in head and neck–shoulder musculature reproduced the pain pattern in the orofacial region in women with myofascial TMD or FMS. Women with myofascial TMD exhibited more active TrPs than women with FMS, whereas women with FMS exhibited larger referred pain areas for neck muscle TrPs. The referred pain areas of temporalis and masseter muscle TrPs were located more inferior (in the orofacial area) in TMD, whereas upper trapezius TrP referred pain was located more inferior (in the neck area) in FMS. The current study supports that generators in different muscles can be involved in orofacial pain (TMD) and neck–face pain associated to FMS.
